# Effects of Different Drying Methods on Drying Characteristics, Microstructure, Quality, and Energy Consumption of Apricot Slices

**DOI:** 10.3390/foods13091295

**Published:** 2024-04-23

**Authors:** Qiaonan Yang, Xiaokang Yi, Hongwei Xiao, Xufeng Wang, Lin Liu, Ziya Tang, Can Hu, Xibing Li

**Affiliations:** 1 College of Mechanical and Electrical Engineering, Fujian Agriculture and Forestry University, Fuzhou 350002, China; 2221298007@fafu.edu.cn (Q.Y.); 22312098006@fafu.edu.cn (L.L.); 2 College of Mechanical and Electrical Engineering, Tarim University, Alar 843300, China; yxk@taru.edu.cn (X.Y.); wxf@taru.edu.cn (X.W.); 3 College of Engineering, China Agricultural University, Beijing 100080, China; xhwcaugxy@163.com; 4 Academy for Advanced Interdisciplinary Studies, Nanjing Agricultural University, Nanjing 210095, China; tang@stu.njau.edu.cn

**Keywords:** apricot, drying method, nutritional characteristics, antioxidant activity

## Abstract

An appropriate drying method is crucial for producing high-quality dried apricots. In this study, the effects of four drying methods, hot air drying (HAD), infrared drying (IRD), pulse vacuum drying (PVD), and vacuum freeze-drying (VFD), on the drying kinetics and physical and nutritional characteristics of apricot slices were evaluated. PVD required the shortest time (16.25 h), followed by IRD (17.54 h), HAD (21.39 h), and VFD (34.64 h). VFD resulted in the best quality of apricot slices, with the smallest color difference (ΔE = 13.64), lowest water activity (0.312 ± 0.015) and browning degree (0.35), highest color saturation (62.84), lowest hardness (8.35 ± 0.47 N) and shrinkage (9.13 ± 0.65%), strongest rehydration ability (3.58 ± 0.11 g/g), a good microstructure, and high nutrient-retention rates (ascorbic acid content: 53.31 ± 0.58 mg/100 g, total phenolic content: 12.64 ± 0.50 mg GAE/g, and carotenoid content: 24.23 ± 0.58 mg/100 g) and antioxidant activity (DPPH: 21.10 ± 0.99 mmol Trolox/g and FRAP: 34.10 ± 0.81 mmol Trolox/g). The quality of PVD-treated apricot slices was second-best, and the quality of HAD-treated apricot slices was the worst. However, the energy consumption required for VFD was relatively high, while that required for PVD was lower. The results of this study provide a scientific basis for the large-scale industrial production of dried apricots.

## 1. Introduction

Apricot is a deciduous tree belonging to the genus *Prunus* (family: Rosaceae). Its fruit is rich in carotenoids, phenols, total flavonoids, and other compounds and has anti-inflammatory, anticancer, and antioxidant properties. It can also reduce the incidence of cardiovascular diseases and certain cancers. Thus, apricot fruit has beneficial effects on human health [1,2]. It is popular among consumers for its bright color, soft texture, and delicious taste [3]. However, it is prone to decay during storage due to its high water content, high respiratory intensity, thin skin, and soft tissue, severely affecting the quality and economic value of the fruit [4]. Drying can reduce the moisture content and water activity of the fruit, prolong its shelf life, and reduce the cost of storage and transport. Commonly used drying methods for agricultural products include natural drying, hot-air drying (HAD), vacuum freeze-drying (VFD), infrared drying (IRD), and pulsed vacuum drying (PVD) [5]. However, high temperatures and long drying times may cause changes in product color, appearance, structure, and texture, as well as loss of nutrients, thereby affecting product quality and limiting its development [6]. Therefore, the effect of drying method on the quality of the final product must be investigated.

In China and other developing countries, the natural drying method is often used for drying apricot fruits and other agricultural products because it is simple and low-cost. However, the disadvantages of natural drying are the long drying time (8–10 days), uncontrollable drying environment, and susceptibility to insects, birds, dust, and other factors, which severely affect the quality of dried apricots [7]. Therefore, to augment the quality of dried apricots and shorten the drying time, mechanical drying technology must be used, instead of traditional natural drying methods. HAD is the most commonly used method for drying and offers the advantages of convenient operation and low cost. Nevertheless, drying at a high temperature for a long period affects the color, texture, rehydration, and heat-sensitive nutrient retention rate of the product. Karabulut et al. [8] explored the effects of different HAD temperatures on the color and carotenoid content of apricots. They found that the drying time was a crucial factor affecting the carotenoid content of dried apricots; an increase in temperature led to color deterioration, but effectively augmented the retention rate of carotenoids. IRD has good heating uniformity and generates products of good quality, but its penetration is limited [9]. Karatas et al. [10] investigated the effect of IRD on the drying characteristics of apricot slices. As the infrared power increases, the drying time of apricot slices decreases and the drying efficiency improves. VFD generates a better-quality product with better color and nutrient retention. Jin et al. [11] found that VFD exhibits efficient dehydration of apricot fruit with relatively low ascorbic acid loss and strong rehydration, but it involves a high cost and long drying time. Therefore, an efficient, energy-saving, controllable, and safe drying method is required to obtain high-quality dried apricots.

PVD is a novel drying method. In this method, the drying chamber was in a cyclic state of vacuum–atmospheric pressure pulsation, which can modify the ultrafine structure of the material and reduce the resistance of water migration and diffusion. Compared with constant vacuum drying, PVD can break the balance between the material and drying medium, and promote the entry of dry air in the material to adsorb and remove the water through the vacuum [12]. Additionally, because the material is mostly in a low-temperature vacuum state during PVD, the anoxic processing environment reduces adverse chemical reactions, effectively inhibits oxidative browning and degradation of heat-sensitive substances, and better retains the material color and nutrients [13]. Compared with constant-pressure vacuum drying, in PVD, the vacuum state reduces the boiling point of the material and modifies the phase transition point of the material temperature, which is beneficial for saving energy. PVD technology is relatively mature and has been successfully used for drying mango slices [14], grapes [15], persimmons [16], wolfberry [17], and garlic slices [18].

The effects of different drying methods on the color, texture characteristics, rehydration, microstructure, nutritional components, and antioxidant activity of apricots have not been systematically and comprehensively investigated. Therefore, this work investigated and compared the effects of PVD, HAD, IRD, and VFD on the drying characteristics, physical properties (color, water activity, hardness, and shrinkage), rehydration, microstructure, nutritional properties (ascorbic acid, total phenols, and carotenoids), antioxidant activity (DPPH and FRAP free radical scavenging ability), and energy consumption of apricot fruits. The purpose was to determine a suitable drying technology for the efficient processing of apricots and to develop dried apricots with high commercial value. The results of this work can provide a basis for the large-scale industrial production of high-quality dried apricots.

## 2. Materials and Methods

### 2.1. Materials

**Figure 1:**
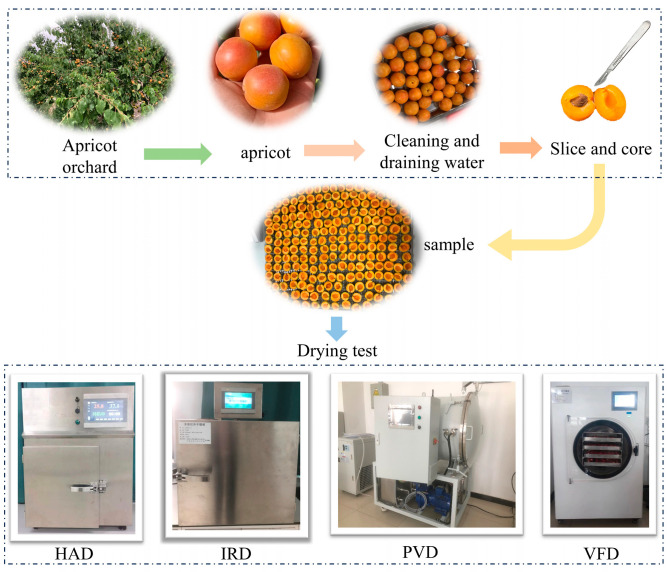
Sample pretreatment and test equipment.

The test sample was *Diaogan apricots* (*Prunus armeniaca* L. cv. Diaoganxing), picked from orchard No. 6, Chang’an Town, Alar City, Xinjiang Uygur Autonomous Region. In order to ensure the source and uniformity of materials, the picking period started on 5 July 2023. Fresh Diaogan apricots of the same size (3.15 ± 0.27 cm) without mechanical damage, insect damage, or disease were selected. Then, the samples were placed in a refrigerator with constant temperature and humidity (4 ± 1 °C, 90% RH) before use. The apricots were pretreated before the drying test (Figure 1). Each apricot was removed from cold storage and maintained for 60 min at room temperature (25 ± 1 °C). The apricot stalk was then removed and cleaned, and the water on the surface of the peel was drained. The apricot was cut radially using a 304 stainless-steel scalpel to form a pair of half-apricot slices. The slices were placed on a 304 stainless-steel material tray for use. The initial moisture content of the fresh samples was 87.15 ± 0.38%.

### 2.2. Drying Method

The fresh apricot slices were dried using HAD, IRD, PVD, and VFD methods until the moisture content was <15% (wet basis). The energy consumption ratio, hardness, color, rehydration, nutritional characteristics, and antioxidant activity of fresh apricot slices and dried apricot slices were compared. The hot air dryer, infrared dryer, vacuum freeze dryer, and pulsating vacuum dryer required for the tests were located in the Key Laboratory of Modern Agricultural Engineering of Tarim University, Alaer City, Xinjiang Uygur Autonomous Region. The optimal drying parameters for each drying technology in this article are based on the results of preliminary experiments.

#### 2.2.1. Hot Air Drying

**Figure 2:**
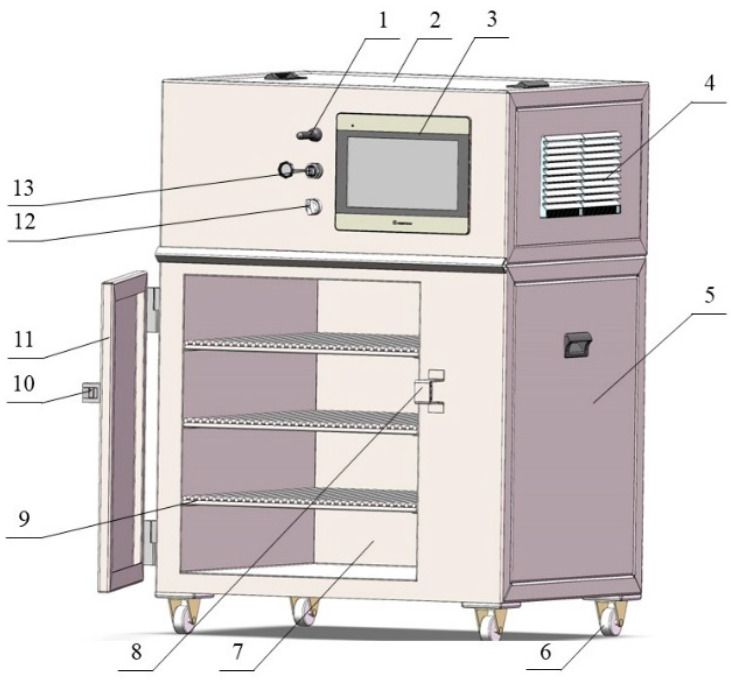
Schematic representation of hot air dryer: 1. signal receiver; 2. electric control box; 3. touch screen; 4. radiating shutter; 5. heating room; 6. support wheel; 7. drying room; 8. lock hook; 9. material plate; 10. door handle; 11. door; 12. power switch; 13. USB socket.

The hot air dryer (fabricated by the College of Engineering of China Agricultural University, Beijing, China) consisted of a touch screen, U-shaped heating pipe, wind speed sensor, temperature and humidity sensor, material rack, material tray, dehumidification fan, spoiler fan, and variable wind speed controller (Figure 2). The material tray containing apricot slices was placed in the hot air dryer and dried at 60 °C and a wind speed of 1.5 m/s.

#### 2.2.2. Infrared Drying

**Figure 3:**
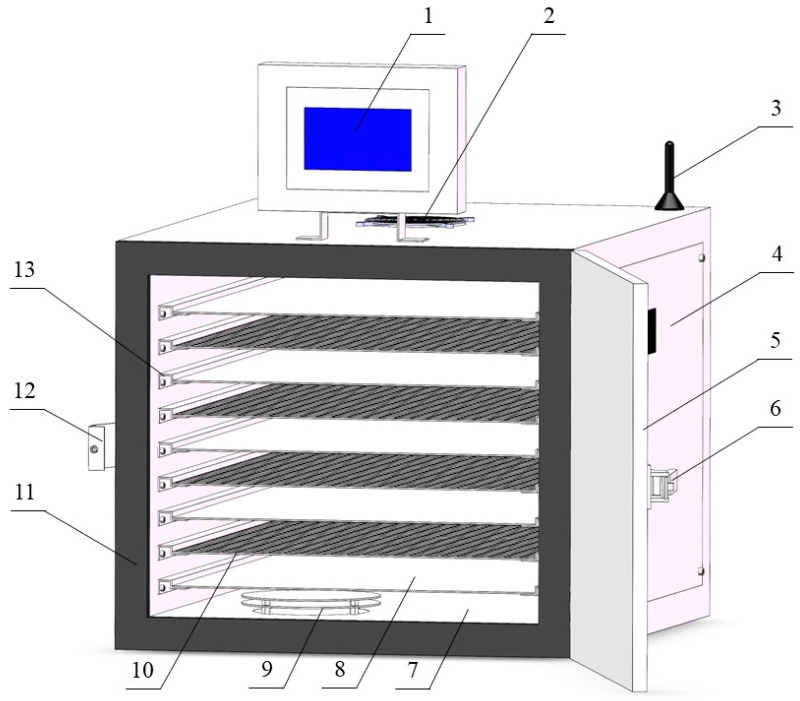
Schematic representation of infrared dryer: 1. touch screen; 2. dehumidification fan; 3. signal receiver; 4. electric control box; 5. door; 6. door handle; 7. drying room; 8. carbon brazing infrared radiation heating plate; 9. inlet; 10. material tray; 11. sealing strip; 12. lock; 13. material rack.

The infrared dryer (fabricated by the College of Engineering of China Agricultural University) consisted of a carbon brazing infrared radiation heating plate, dehumidification fan, material rack, material tray, touch screen, and temperature and humidity sensor (Figure 3). The material tray containing the apricot slices was placed in the infrared dryer. The temperature of the carbon brazing heating plate in the drying chamber was set to 60 °C, and the radiation power was set to 1.1 kW/m^2^ for drying. The size of the carbon fiber infrared radiation heating plate was 500 × 600 mm, the infrared wavelength was 5–15 μm, the surface temperature was 90 °C, and the carbon fiber infrared radiation heating plate was 50 mm away from the material tray.

#### 2.2.3. Vacuum Freeze-Drying

**Figure 4:**
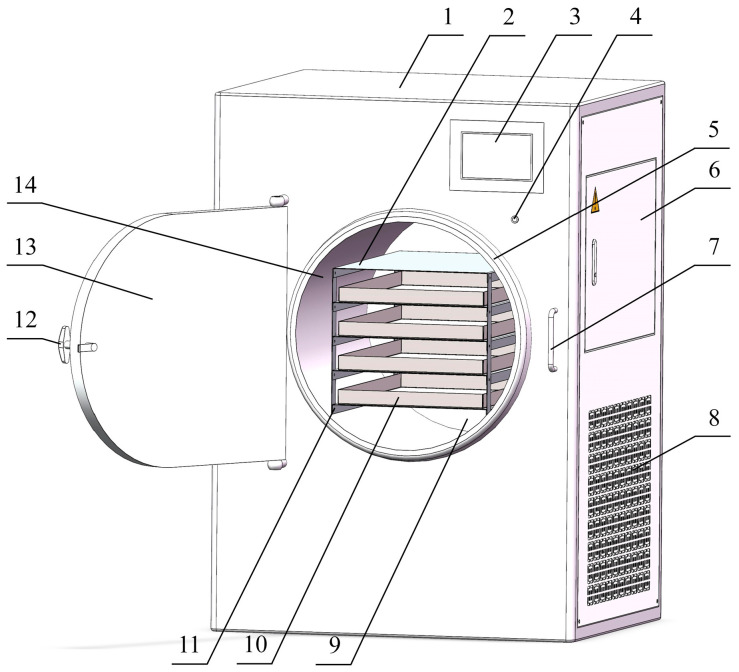
Schematic representation of vacuum freeze-drying machine: 1. casing; 2. heating plate; 3. touch screen; 4. power switch; 5. sealing ring; 6. electric control box; 7. lock; 8. heat dissipation window; 9. cold trap room; 10. material tray; 11. material rack; 12. door handle; 13. door; 14. water catcher.

The vacuum freeze-dryer (self-fabricated by the School of Mechanical and Electrical Engineering of Tarim University, Xinjiang, China) consisted of a cooling chamber, material rack, heating plate, material tray, temperature and humidity sensor, compressor, heat exchanger, and touch screen (Figure 4). The apricot slices on the material tray were pre-frozen at −20 °C for 24 h and then quickly transferred to the vacuum freeze dryer. The slices were dried at a vacuum pressure of 60 Pa. Using the touch screen, the cooling temperature and heating plate temperature were set to −40 °C and 45 °C, respectively.

#### 2.2.4. Pulsed Vacuum Drying

**Figure 5:**
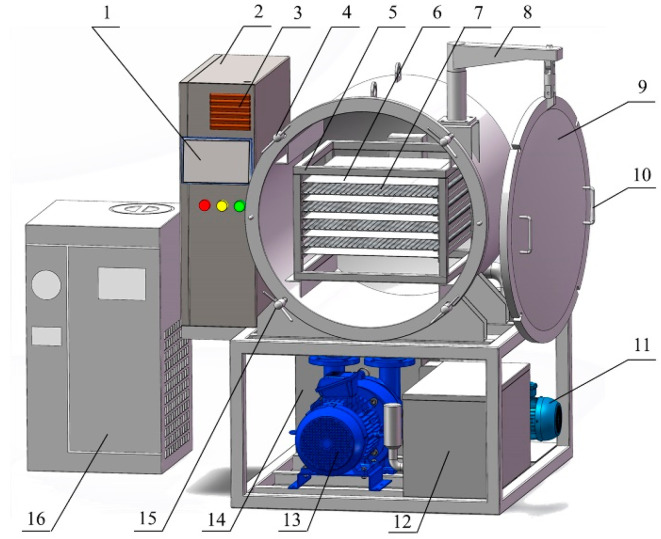
Schematic representation of the pulse vacuum dryer: 1. touch screen; 2. electric control box; 3. heat dissipation window; 4. lock; 5. material frame; 6. heating plate; 7. material plate; 8. hanging arm; 9. door; 10. door handle; 11. circulating water pump; 12. constant temperature inlet box; 13. vacuum pump; 14. constant temperature water tank; 15. rack; 16. refrigeration system.

The pulsating vacuum dryer (self-fabricated by the School of Mechanical and Electrical Engineering of Tarim University) consisted of a cooling chamber, material rack, heating plate, material tray, temperature and humidity sensor, compressor, heat exchanger, and touch screen (Figure 5). The apricot slices on the material plate were placed in the dryer. According to preliminary experimental results, the parameters were set to a drying temperature of 60 °C and a normal pressure/vacuum ratio of 3 min:12 min. The cycle was repeated every 15 min, and the pressure in the drying chamber under the vacuum was 6.0 kPa.

### 2.3. Drying Kinetics

The moisture ratio (*MR*) of apricot slices was calculated as shown in Equation (1).

(1) $$MR=\frac{m_{t}-m_{e}}{m_{0}-m_{e}}$$

where $m_{0}$ is the initial dry basis moisture content of apricot slices (g/g), $m_{t}$ is the dry basis moisture content of apricot slices at time *t* (g/g), and $m_{e}$ is the dry basis moisture content of apricot slices at equilibrium (g/g).

The drying rate (*DR*) of apricot slices was calculated as shown in Equation (2) [12].

(2) $$DR=\frac{m_{t1}-m_{t2}}{t_{2}-t_{1}}$$

where $t_{1}$ and $t_{2}$ indicate the drying times of apricot slices (h), and $m_{t1}$ and $m_{t2}$ are the dry-basis moisture contents at $t_{1}$ and $t_{2}$ respectively (g/g).

### 2.4. Energy Consumption Ratio

The energy consumption ratio refers to the energy consumed to remove moisture per unit mass. The energy consumed for drying the apricot slices under different drying methods was recorded using an electric meter (Model: DTSY631, Zhejiang Tiantian Technology Co., Ltd., Wenzhou, China). Water loss of the slices was determined using the weighing method. The energy consumption ratio (*E*) was calculated as shown in Equation (3) [12].

(3) $$E=\frac{Q}{m}$$

where *E* is the unit energy consumption ratio (kJ·h/kg); *Q* is the energy consumption measured using the electric meter before and after drying (kJ·h); and *m* is the total amount of dehydration of apricot slices during drying (kg).

### 2.5. Color

A portable spectrophotometer (Model: DS-700D, Hangzhou Color Spectrum Technology Co., Ltd., Hangzhou, China) was used to determine the color of the apricot slices before and after drying. Color is represented by *L*, *a*, and *b*, where *L* is the brightness value, *a* is the red and green value, and *b* is the blue and yellow value. During color measurement, the portable spectrophotometer was calibrated in black and white. The calculation equation of color saturation (*C*) is shown in Equation (4) and the calculation equation of color difference (∆*E*) is shown in Equation (5) [12].

(4) $$C=\sqrt{a^{2}+b^{2}}$$

(5) $$∆E=\sqrt{{\left(L-L_{1}\right)}^{2}+{\left(a-a_{1}\right)}^{2}+{\left(b-b_{1}\right)}^{2}}$$

where *L*, *a*, and *b* represent the color parameters of the original fresh apricot slices, and $L_{1},{\;a}_{1},$ and $b_{1}$ represent the color parameters of the dried apricot slices.

A slightly modified version of the method described by Chu et al. [19] was used to determine the degree of browning of dried apricots. A 0.2 g sample was placed in a mortar. An appropriate amount of 2% acetic acid solution was added to the mortar and the sample was fully ground. The ground mixture was added to a 50 mL centrifuge tube for 120 min and centrifuged at 8000 rpm in a refrigerated centrifuge for 20 min. The supernatant was extracted, and 0.2% acetic acid solution was used as a blank control. Absorbance was measured at 420 nm using an ultraviolet spectrophotometer. The browning degree is expressed as the unit of absorbance per gram of sample (AU/g).

### 2.6. Water Activity

The water activity of the fresh apricot slices and dried apricot slices was measured using an intelligent water activity-measuring instrument (Model: HD-3A, Wuxi Huake Instrument Co., Ltd., Wuxi, China).

### 2.7. Hardness

The hardness of the dried apricot slices was determined using a texture analyzer (TA-XT plus, Stable Micro Systems, Surrey, UK) according to the method of Yang et al. [2]. The side with the apricot peel was placed on the test platform, and a 50 mm diameter cylindrical probe was used for the measurement. The measurement speed was set to 2 mm/s; the pre-measurement and post-measurement speeds were 1 and 5 mm/s, respectively; the strain applied was 30%; the trigger was 10 g; the measurement time was 5 s; and the probe performed two measurements. The form of trigger used was to conduct a rapid compression test on the dried apricot slices to determine hardness.

### 2.8. Shrinkage

During drying, the sample structure collapses due to water loss, manifested as volume shrinkage. Therefore, sample shrinkage was determined using the method described by Yang et al. [20] after slight modifications. A 100 mL beaker was filled with millet, and a portion of millet was added to a 50 mL measuring cylinder. The fresh apricot slices and dried apricot slices were placed into separate beakers, and the millet in the measuring cylinder was added to the 100 mL beaker. The remaining millet in the measuring cylinder was obtained as the volume of fresh apricot slices and dried apricot slices. The shrinkage rate (*VR*) was calculated as shown in Equation (6) [20].

(6) $$VR=\left(1-\frac{V_{t}}{V}\right)\times 100\%$$

where *V* is the volume of fresh apricot slices (${cm}^{3}$) and $V_{t}$ is the volume of dried apricot slices (${cm}^{3}$).

### 2.9. Rehydration Ratio

The dried apricot slices were placed in a beaker of distilled water, and the rehydration test was conducted in a constant-temperature water bath at 30 °C. The dried apricot slices were removed from the beaker and weighed every hour. Before the sample was weighed, the water on the sample surface was removed using absorbent paper. After the sample was weighed, it was put back into the original beaker, and the aforementioned steps were repeated until the sample weight reached a constant value. The rehydration ratio (*RR*) was calculated as shown in Equation (7) [20].

(7) $$RR=\frac{W_{t}}{W}\times 100\%$$

where *W* is the initial weight of dried apricot slices (g) and $W_{t}$ is the weight of dried apricot slices after rehydration (g).

### 2.10. Microstructure

A scanning electron microscope (Model: SU3500, Hitachi High-Tech (Shanghai) Co., Ltd., Tokyo, Japan) was used to observe the microstructure of dried apricot slices. The dried sample was placed on a conductive adhesive and sprayed with gold, and the images were observed at 100× magnification under an accelerating voltage of 10 kV.

### 2.11. Nutritional Characteristics

#### 2.11.1. Ascorbic Acid Content

Based on a slightly modified version of the method described by Jiang et al. [21], 2,6-dichloroindophenol sodium was used for determining the ascorbic acid content of the samples. A 2 g sample was placed in a mortar, ground into a paste with 2% (*w*/*w*) oxalic acid solution, diluted with 1% (*w*/*w*) oxalic acid solution in a 100 mL volumetric flask, and shaken well. After allowing the mixture to stand for 2 h, it was transferred to a 20 mL centrifuge tube and centrifuged at 6000 rpm for 30 min in a refrigerated centrifuge. Then, 10 mL of the supernatant was titrated using the 2,6-dichloroindophenol sodium reagent until the solution turned pink and did not fade within 15 s. The ascorbic acid content of the sample, expressed as *H*, was calculated using Equation (8) [21].

(8) $$H=\frac{100\left[{\left(V-V_{1}\right)}^{t}\right]}{m}$$

where *H* is the ascorbic acid content, mg/100 g; *t* is 0.02 mg/mL standard vitamin C solution per milliliter of 2,6-dichloroindophenol sodium; *V* is the volume of 2,6-dichloroindophenol sodium consumed; $V_{1}$ is blank; and *m* is the sample mass, g.

#### 2.11.2. Total Phenol Content

A slightly modified version of the method described by An et al. [22] was used to prepare the total phenol extract. A 1 g sample was placed in a mortar and ground with an appropriate amount of 80% methanol solution. The fully ground and mixed solution was stored in the dark for 24 h, extracted under 40 kHz ultrasonic conditions for 60 min, placed in a 50 mL centrifuge tube, and centrifuged at 8000 rpm for 20 min in a refrigerated centrifuge. The supernatant was collected. Referring to the methods of Chen et al. [23], the total phenol content of the samples was determined using a slightly modified version of the Folin–Ciocalteu method. Briefly, 0.2 mL of the supernatant and 1 mL of Folin–Ciocalteu reagent was diluted with distilled water and added to a 20 mL volumetric flask. The solution was shaken for 10 min, and 3 mL of 20 g/L sodium carbonate solution was added. The solution was maintained at 25 °C for 2 h, and the sample absorbance was measured at 765 nm using an ultraviolet spectrophotometer. The results are expressed as gallic acid equivalent, mg GAE/g d.b.

#### 2.11.3. Total Carotenoid Content

A modified version of the method described by Juhnevica-Radenkova et al. [24] was used for determining the total carotenoid content of the samples. A 2 g sample was placed in a mortar. Then, 50 mL of the extract (n-hexane:acetone:methanol = 2:1:1, *v*/*v*/*v*) was added to the sample in batches and ground completely. The mixture was stirred at 300 rpm for 60 min using a magnetic stirrer at 30 °C, transferred to a centrifuge tube, and centrifuged at 8000 rpm for 30 min. Then, 50 mL of the supernatant was collected. Absorbance was measured at 450 nm using an ultraviolet spectrophotometer. The results are expressed as β-carotene equivalent, mg β-carotene/100 g d.b.

### 2.12. Determination of Antioxidant Activity

Following the methods described by An et al. [22], the total phenolic extract of the sample was prepared. The antioxidant capacity of the sample was determined by evaluating its scavenging capacity for 1,1-diphenyl-2-picrylhydrazyl (DPPH) free radical and its ferric-reducing antioxidant power (FRAP).

#### 2.12.1. DPPH Determination

The method of Juhnevica-Radenkova et al. [24] with slight modifications was used to determine the *DPPH* free radical scavenging capacity of the sample. First, 5 mL of the extract was collected and transferred to the test tube. Then, 5 mL of 0.1 mmol/L *DPPH* solution was added to the test tube and fully shaken. Subsequently, the mixture was heated in a 25 °C constant-temperature water bath for 30 min in the dark. Absorbance was measured at 517 nm using an ultraviolet spectrophotometer. The results are expressed as Trolox equivalent, mmol Trolox/g d.b. The total antioxidant capacity is expressed as *DPPH*, as shown in Equation (9).

(9) $$DPPH=\left(A_{1}-A_{2}+0.0081\right)÷1.14144\times V_{1}÷\left(V_{1}÷V_{2}\times W\right)$$

where $A_{1}$ is the absorbance value of the blank sample; $A_{2}$ is the absorbance value of the measured sample; $V_{1}$ is the volume of the sample in the reaction, μL; $V_{2}$ is the volume of the added extraction solution, mL; and *W* is the mass of the sample, g.

#### 2.12.2. FRAP Determination

By referring to the methods of Yan et al. [25], fresh FRAP reagent was prepared by completely mixing 10 mmol/L 2,4,6-tripyridyltriazine solution, 300 mmol/L sodium acetate (pH 3.6), and 20 mmol/L FeCl_3_ 6H_2_O in a 1:10:1 volume ratio. Then, 0.1 mL of the phenolic extract of the sample was collected and transferred to a test tube. Later, 4.9 mL of the fresh FRAP reagent was added to the test tube and mixed well. The mixture was heated in a 37 °C constant-temperature water bath for 10 min in the dark. Absorbance was measured at 593 nm using an ultraviolet spectrophotometer. The results are expressed as Trolox equivalent, mmol Trolox/g d.b. The total antioxidant capacity was expressed as FRAP, as shown in Equation (10).

(10) $$FRAP=\left(A_{3}-A_{4}-0.0134\right)÷2.4832\times V_{3}÷\left(V_{3}÷V_{4}\times W\right)$$

where $A_{3}$ is the absorbance value of the measured sample; $A_{4}$ is the absorbance value of the blank sample; $V_{3}$ is the volume of the sample in the reaction, μL; $V_{4}$ is the volume of the added extraction solution, mL; and *W* is the mass of the sample, g.

### 2.13. Data Processing

All experiments were repeated three times, and the results were expressed as the mean ± standard deviation. Excel software (version 2021, Microsoft Inc., Redmond, WA, USA) was used to organize experimental data. SPSS software (Version 27.0, SPSS Inc., Chicago, IL, USA) was used for one-way ANOVA and Duncan’s tests to analyze the effects of different drying methods on the drying time, color, water activity, hardness, shrinkage rate, rehydration ability, nutritional components, antioxidant capacity, and energy consumption of dried apricot slices (*p* < 0.05). Origin software (version 2021, OriginLab Inc., Northampton, MA, USA) was used to plot the resulting images.

## 3. Results and Discussion

### 3.1. Effects of Different Drying Methods on the Drying Time and Energy Consumption Ratio of Apricot Slices

**Figure 6:**
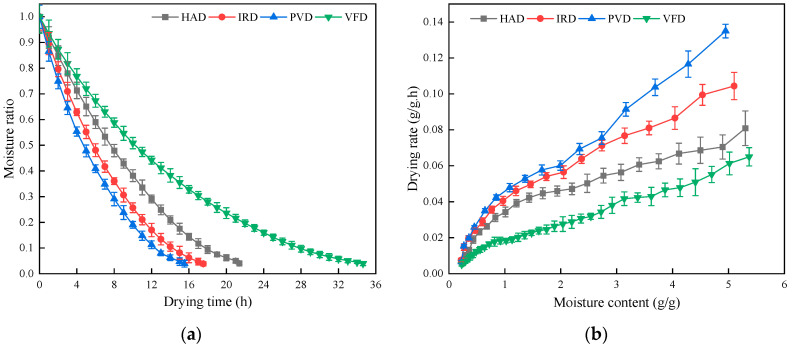
Drying curves and drying rate curves of apricot slices with different drying methods: (**a**) relationship between drying time and moisture ratio; (**b**) relationship between drying rate and moisture content (dry basis).

The effects of different drying methods on the moisture ratio and drying rate of apricot slices are shown in Figure 6. There were significant differences in drying kinetics among drying methods. As shown in Figure 6a, the moisture content of apricot slices decreased as the drying time increased, which is a common phenomenon in the processing of agricultural products [12,26]. The times required for HAD, IRD, PVD, and VFD were 21.39 h, 17.54 h, 15.53 h, and 34.64 h, respectively. Compared with the drying time required for VFD, the times required for HAD, IRD, and PVD were shortened by 38.25%, 49.36%, and 55.17%, respectively. These results showed that the time required to dry apricot slices varied according to the drying method, with the longest time required for VFD and the shortest time for PVD. Therefore, PVD is more conducive to shortening the drying time of apricots.

The relationship between the drying rate of apricot slices and the moisture content (dry basis) is shown in Figure 6b. PVD had a higher drying rate compared with HAD, IRD, and VFD. It is possible that PVD can change the ratio of atmospheric pressure to vacuum in the drying chamber by circulation, disturbing the air in the drying chamber to accelerate the evaporation and diffusion of the sample water [27]. However, holding the sample in a vacuum state can reduce the boiling point of water (the corresponding boiling point of water when the pressure is 6.0 kPa is 85.93 °C.), change the intercellular pores of the sample, and promote the migration of internal moisture to the surface of the material, thereby reducing the drying time [28]. As shown in Figure 6b, the drying rate of VFD apricot slices was the lowest, which may be explained by the low-temperature environment, the reduced heat transfer efficiency by the pre-freezing treatment, and the low migration rate of internal moisture to the surface.

### 3.2. Effects of Different Drying Methods on the Color of Apricot Slices

Color is a crucial indicator of fruit drying quality and affects the market value and consumer acceptance. However, different drying methods have diverse effects on the color of apricot slices (Table 1). The brightness (*L*) values of the HAD-, IRD-, and PVD-treated apricot slices were significantly reduced, and those of the HAD-treated slices were the lowest (43.56 ± 2.16). A long drying time at a higher temperature leads to the excessive consumption of anti-browning substances, which causes a decrease in the brightness (*L*) of apricot slices. Yellowness (*b*) is a critical index that indicates the browning degree of apricot slices after drying. Yellowness (*b*) was highest for the VFD-treated apricot slices (58.07 ± 1.54) and lowest for HAD-treated apricot slices (33.23 ± 3.19). The Maillard reaction, caramelization reaction, and ascorbic acid oxidative decomposition may occur during drying at a high temperature. These reactions destroy the natural color of the apricot slices and cause non-enzymatic browning [29]. The color difference (Δ*E*) values for the PVD- and VFD-treated apricot slices were 14.89 ± 0.50 and 13.64 ± 0.55, respectively, and those for the HAD- and IRD-treated apricot slices were larger (26.45 ± 1.36 and 18.72 ± 0.41, respectively) which may be related to the drying temperature. The color saturation (*C*) values of the VFD-, PVD-, and HAD-treated apricot slices were 62.84 ± 1.87, 58.15 ± 0.39, and 38.52 ± 3.06, respectively. Higher color saturation (*C*) indicates a better color. Compared with the observations for HAD and IRD, PVD and VFD resulted in better retention of the original color of apricot slices. This was possibly due to the low temperature and vacuum environment used in these methods [28], which inhibited the enzymatic browning reaction and pigment destruction [30]. The browning degree of the VFD-treated apricot slices was the lowest (0.35 ± 0.04 AU/g), and that of the PVD-treated slices was 0.50 ± 0.08 AU/g. Therefore, high-temperature drying causes the browning reaction and affects the quality of apricot slices. Other studies have reported similar results. Wang [31] and Baysal et al. [32] noted that the colors of IRD-treated mushrooms, carrots, and garlic were better than those of HAD-treated samples, and VFD led to better retention of the color of fresh samples.

### 3.3. Effects of Different Drying Methods on the Water Activity of Apricot Slices

**Figure 7:**
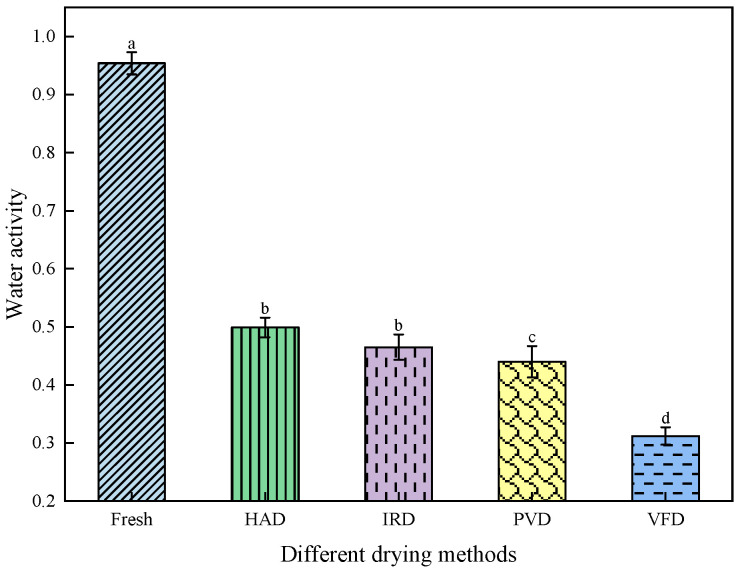
Effects of different drying methods on the water activity of apricot slices. Values with different letters in each column are considered significantly different (*p* < 0.05).

Water activity is a crucial index affecting the storage stability of dried fruits. When the water activity of dried fruits is <0.6, microbial growth and chemical reactions are effectively inhibited [22]. As the water content of fresh apricots is high, their respiration intensity is high and water activity is relatively high (i.e., 0.954 ± 0.019). The water activities of apricot slices dried through HAD, IRD, PVD, and VFD decreased to 0.499 ± 0.177, 0.485 ± 0.022, 0.440 ± 0.027, and 0.312 ± 0.015, respectively (Figure 7). The water activity of dried apricot slices meets the standard for safe storage. The water activity of the sample is positively correlated with the browning degree. If the browning degree is higher, the water activity is greater. During VFD and PVD, apricot slices were in a low-temperature vacuum state; therefore, the anoxic processing environment reduced adverse chemical reactions, inhibited the oxidation reaction, better prevented browning, and reduced water activity [33]. However, the ambient temperature during VFD was lower than that during PVD, which effectively maintained the porous structure of the apricot slices [34], increased their hydrophobicity, and reduced their water activity. Kręcisz et al. [35] found similar results while drying zucchini slices. In summary, the water activity was lowest for the VFD-treated apricots, followed by PVD-, HAD-, and IRD-treated apricots. Therefore, the storage stability and safety of the PVD- and VFD-treated apricot slices were relatively high.

### 3.4. Effects of Different Drying Methods on the Hardness and Shrinkage of Apricot Slices

**Figure 8:**
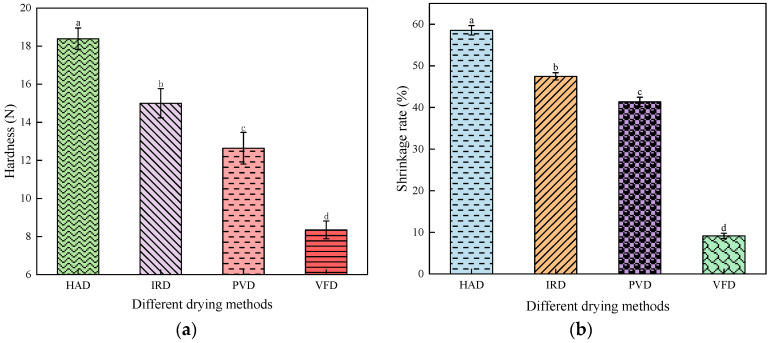
Effects of different drying methods on the hardness and shrinkage of apricot slices: (**a**) hardness; (**b**) shrinkage ratio. Values with different letters in each column are considered significantly different (*p* < 0.05).

Sample shrinkage is a common physical phenomenon during the drying of agricultural products. Hardness is a critical indicator of material properties [36]. The hardness and volume shrinkage of the dried apricot slices are presented in Figure 8. The hardness and volume shrinkage of the HAD-treated apricot slices (18.39 ± 0.56 N and 58.55 ± 1.14%, respectively) were significantly higher than those of the IRD-, PVD-, and VFD-treated slices (*p* < 0.05), reflecting rapid water loss and the continuous separation of macromolecules during HAD. Zhao et al. [37] reported similar results for Acanthopanax senticosus. The hardness and volume shrinkage of the VFD-treated apricot slices were the lowest (8.35 ± 0.47 N and 9.13 ± 0.65%, respectively). The internal ice crystals of the VFD-treated apricot slices may slowly sublimate during drying, potentially resulting in less damage and effectively maintaining the structural morphology of the apricot slices [38]. Compared with those of the HAD- and IRD-treated slices, the hardness and volume shrinkage of the PVD-treated apricot slices were relatively small (12.64 ± 0.83 N and 43.36 ± 1.13%, respectively). Although the volume shrinkage and hardness of the VFD-treated apricot slices were the lowest, the drying time was longer and energy consumption was relatively high. PVD can effectively shorten the drying time, improve the hardness of apricot slices, and maintain the appearance of the sample. These results provide a theoretical basis for developing new processes and devices.

### 3.5. Effects of Different Drying Methods on the Rehydration of Apricot Slices

**Figure 9:**
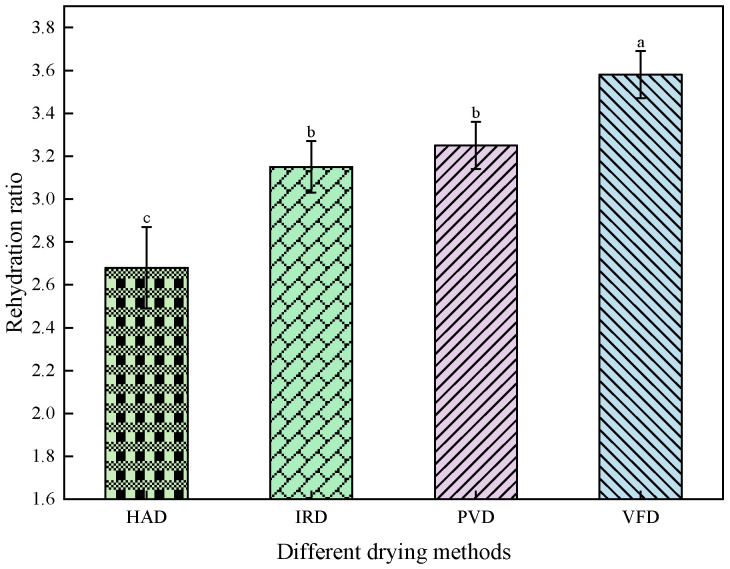
Effects of different drying methods on rehydration of apricot slices. Values with different letters in each column are considered significantly different (*p* < 0.05).

Rehydration is an important index for judging the drying characteristics of samples. The effects of different drying methods on the rehydration ratio of apricot slices are presented in Figure 9. The rehydration ratios of the HAD-, IRD-, PVD-, and VFD-treated apricot slices were significantly different (*p* < 0.05). The HAD-treated apricot slices had the lowest rehydration ratio of 2.68 ± 0.19. This may be due to crusting on the surface and collapse of the internal cell matrix due to high temperatures, thereby affecting the entry of external water into the slices. However, the rehydration ratios of the VFD- and PVD-treated apricot slices were relatively high (3.58 ± 0.11 and 3.25 ± 0.11, respectively). The vacuum environment may be beneficial for retaining the cell structure of the sample and increasing water absorption.

### 3.6. Effect of Different Drying Methods on the Microstructure of Apricot Slices

**Figure 10:**
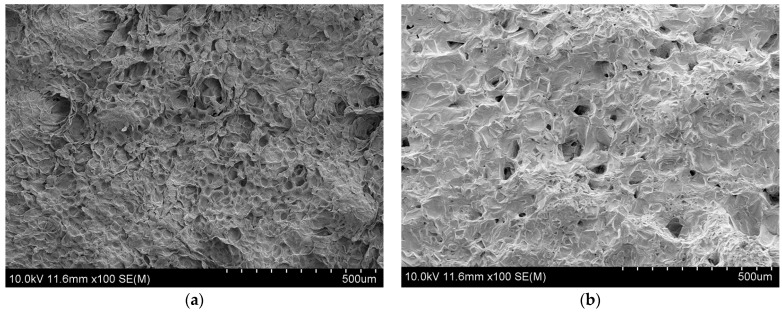

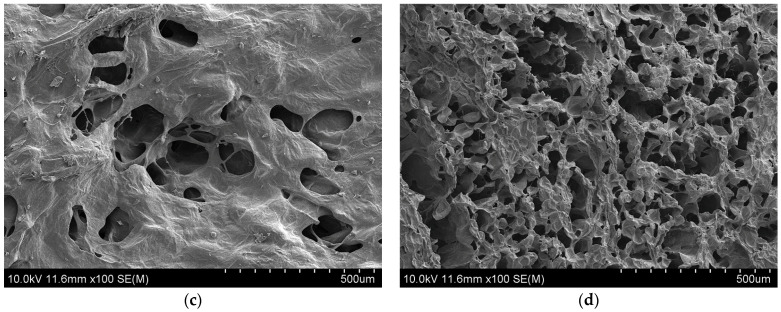
Microstructure of apricot slices dried by different drying methods: (**a**) HAD; (**b**) IRD; (**c**) PVD; (**d**) VFD.

The effects of different drying methods on the microstructure of apricot slices were observed by scanning electron microscopy, as shown in Figure 10. The apricot slices exhibited crusting and cavity collapse during the HAD process, forming a dense structure (Figure 10a). Irregular holes were observed in the apricot slices during the IRD process (Figure 10b). There were large holes in the apricot slices during the PVD process, accompanied by collapse (Figure 10c). The apricot slices had a honeycomb structure and the holes were evenly arranged during the VFD process (Figure 10d). The cell structure of HAD apricot slices collapsed, resulting in the weakest rehydration ability, the largest volume shrinkage, and the highest hardness. Yang et al. [16] reported similar phenomena. In addition, the microstructure of IRD apricot slices showed different regular holes because the infrared short waves could penetrate the surface of the material to transmit internal heat. However, the penetration ability of infrared short waves is limited, resulting in uneven heating. The microstructure of VFD apricot slices was relatively complete, showing a sponge-like porous structure. The frozen water molecule crystals likely sublimated into a gaseous state during the drying process [39], and holes remained in the sample. VFD and pre-freezing better preserved the tissue structure of the sample [40], resulting in the strongest rehydration capacity, minimal volumetric shrinkage, and lowest hardness. The microstructure of PVD apricot slices had many large pores. This may be due to the lower pressure during the drying process leading to a decrease in the boiling point of free water in the apricot slices, resulting in a large vapor difference, which helps to form a porous structure. Xie et al. [41] observed a similar phenomenon during wolfberry drying.

### 3.7. Effects of Different Drying Methods on Ascorbic Acid and Total Phenols in Apricot Slices

**Figure 11:**
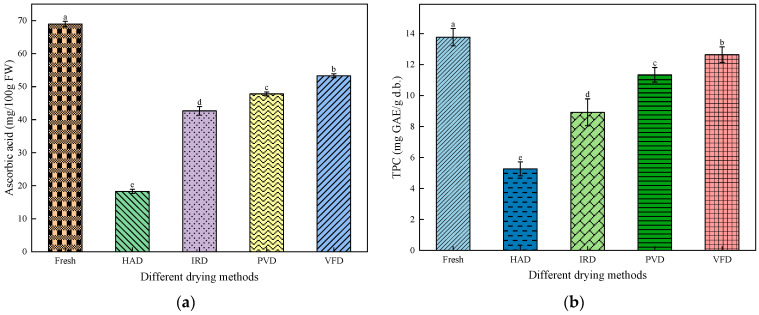

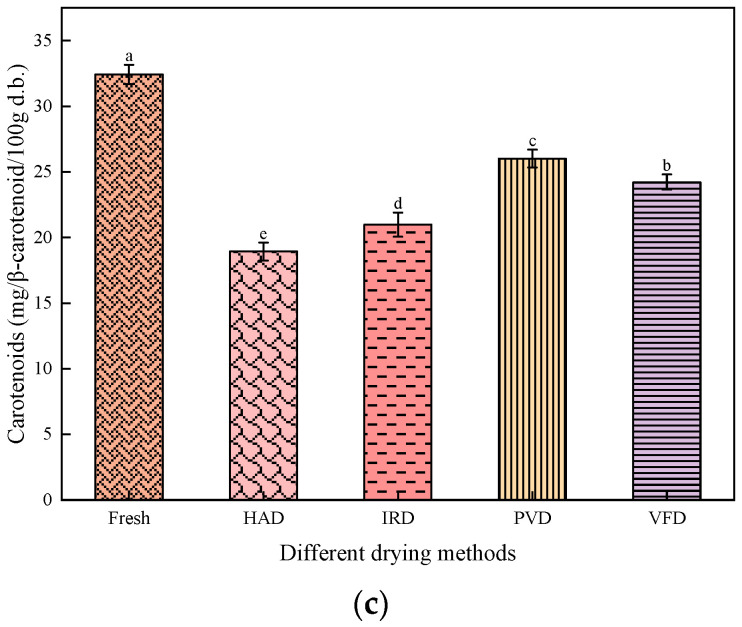
Effects of different drying methods on the nutritional components of apricot slices: (**a**) ascorbic acid content; (**b**) total phenol content; (**c**) carotenoid content. Values with different letters in each column are considered significantly different (*p* < 0.05).

The effects of different drying methods on ascorbic acid and total phenol contents in apricot slices differed significantly (*p* < 0.05) (Figure 11a,b). During drying, phenolic compounds and ascorbic acid were degraded by heat. Therefore, the total phenol and ascorbic acid contents of the apricot slices were significantly lower than those of the fresh samples [42]. The ascorbic acid and total phenol contents of the VFD-treated apricot slices were the highest (53.31 ± 0.58 mg/100 g FW and 12.64 ± 0.50 mg GAE/g, respectively). The total phenol contents of the PVD-, IRD-, and HAD-treated slices were 11.34 ± 0.47 mg GAE/g, 8.92 ± 0.87 mg GAE/g, and 5.27 ± 0.45 mg GAE/g, respectively, indicating that the total phenol content was lowest for HAD-treated apricot slices. Additionally, the trends of the total phenol content in the apricot slices subjected to different drying methods were consistent with trends in the ascorbic acid content. However, the retention rates of phenolic compounds and ascorbic acid in the VFD- and PVD-treated apricot slices were relatively high, indicating that the low-temperature vacuum environment was optimal for retaining the heat-sensitive and easily oxidized components in the sample [43]. High-temperature drying accelerates the degradation of phenolic compounds and ascorbic acid, but it also shortens the sample drying time, which offsets the adverse effects of high-temperature drying. Li [44] and Wang et al. [43] reported similar results while investigating the drying of walnut powder and pepper leaves, respectively. Nutrient retention in the samples was affected by the drying temperature and time.

### 3.8. Effects of Different Drying Methods on Carotenoids in Apricot Slices

Carotenoids are the main pigment source in apricots [45]. Accordingly, changes in the carotenoid content of apricots after drying were analyzed (Figure 11c). The retention rates of carotenoids in the PVD- and VFD-treated apricot slices were relatively high (26.02 ± 0.68 mg β-carotenoid/100 g and 24.23 ± 0.58 mg β-carotenoid/100 g, respectively). The carotenoid content of the PVD-treated apricot slices was higher than that of the VFD-treated apricot slices because the double bonds of carotenoids were easily oxidized [46], and the time required for VFD was longer, which could trigger the oxidation of the carotenoid double bonds in the slices. The carotenoid contents of the IRD- and HAD-treated apricot slices were 20.99 ± 0.92 mg β-carotenoid/100 g and 18.99 ± 0.68 mg β-carotenoid/100 g, respectively. In contrast, the carotenoid content of the HAD-treated apricot slices was the lowest, consistent with the results of Karabulut et al. [8]. The drying method significantly affected the carotenoid content of the apricot slices (*p* < 0.05), with contents of 18.99–26.02 mg β-carotenoid/100 g. During drying, the carotenoid content of the apricot slices decreased significantly, possibly due to a thermal effect on carotenoids [47].

### 3.9. Effects of Different Drying Methods on the Antioxidant Activity of Apricot Slices

**Figure 12:**
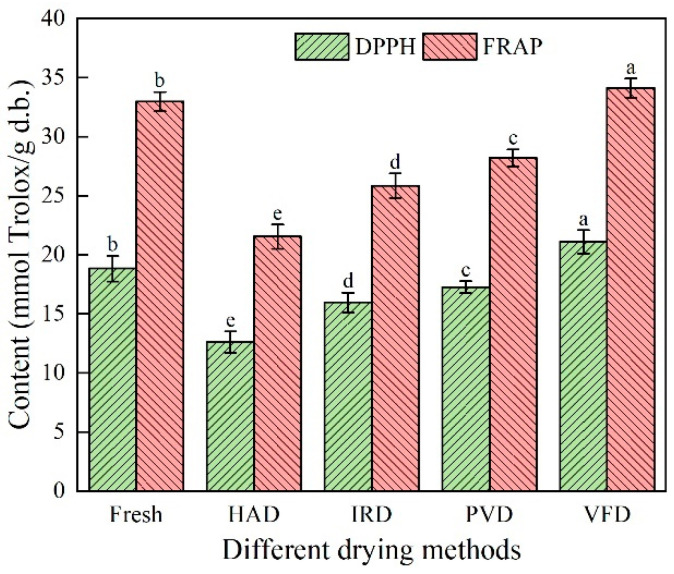
Effects of different drying methods on antioxidant activity of apricot slices. Values with different letters in each column are considered significantly different (*p* < 0.05).

The effects of different drying methods on the antioxidant activity of apricot slices were studied using DPPH and FRAP assays (Figure 12). The DPPH and FRAP free radical scavenging abilities of the apricot slices dried using different drying methods were significantly different from those of the fresh apricot slices (*p* < 0.05). The DPPH and FRAP free radical scavenging abilities of the VFD-treated apricot slices were the strongest (21.10 ± 0.99 mmol Trolox/g and 34.10 ± 0.81 mmol Trolox/g, respectively) and were 12.06% and 3.43% higher than those of the fresh apricot slices. This may have been due to the formation and sublimation of crystals inside the material during VFD, which destroyed the cell structure and increased the extractable antioxidant active substances. Xu [48] and An et al. [22] obtained the same results while studying VFD-treated okra and soybeans. The DPPH and FRAP free radical scavenging abilities of dried samples were stronger than those of fresh samples. The DPPH and FRAP free radical scavenging abilities of the PVD- and IRD-treated apricot slices (DPPH: 17.26 ± 0.51 and 15.97 ± 0.83 mmol Trolox/g; FRAP: 28.19 ± 0.73 and 25.85 ± 1.04 mmol Trolox/g, respectively) were lower than those of the VFD-treated slices, with those of the IRD-treated apricot slices being the weakest (DPPH: 12.60 ± 0.92 mmol Trolox/g; FRAP: 21.54 ± 1.03 mmol Trolox/g, respectively). After the PVD, IRD, and IRD treatments, the DPPH free radical scavenging ability of the fresh apricot slices decreased by 8.34%, 15.19%, and 33.09%, respectively, and their FRAP free radical scavenging ability decreased by 14.50%, 21.60%, and 34.67%, respectively. In summary, the antioxidant activity of the VFD-treated apricot slices was highest, and the retention rates of the total phenolic and ascorbic acid contents of these apricot slices may also have been the highest. The low temperature and vacuum drying environment effectively reduced total phenol, ascorbic acid, and carotenoid degradation in the samples. These findings indicate that phenolic compounds, ascorbic acid, and carotenoids are key substances involved in the antioxidant activity of apricot slices. Similar results were found in ginger [49] and *Lonicera edulis* [50]. Compared with the VFD-treated apricot slices, the antioxidant activity of the HAD-treated slices was lower, which may be explained by the rapid decomposition of phenolic compounds under long-term, high-temperature conditions. Therefore, selecting an appropriate drying method is critical for retaining the antioxidant activity of the sample.

### 3.10. Energy Consumption Analysis

The energy consumed to attain the final dry condition (<15% wet basis) of the apricots differed among drying methods (Table 2). The energy consumption estimates for VFD > HAD > PVD > IRD were 26.88 ± 1.07 kWh/kg, 5.19 ± 0.21 kWh/kg, 4.57 ± 0.41 kWh/kg, and 3.47 ± 0.29 kWh/kg, respectively. The energy consumed during PVD was less than that consumed during HAD; this can be attributed to the longer time required for HAD and the continuous operation of the high-energy-consuming wind-speed centrifuge during drying [51]. The energy consumed during PVD of apricot slices was higher than that consumed by IRD hot air, possibly because a large amount of energy was consumed during the operation of the vacuum pump in the PVD method [36].

## 4. Conclusions

This study comprehensively analyzed the effects of four different drying methods (HAD, IRD, PVD, and VFD) on the drying characteristics, color, water activity, hardness, shrinkage, rehydration, microstructure, nutritional content, and antioxidant capacity of apricots, as well as energy consumption. Several key results were obtained. (1) The four different drying methods had significant effects on the drying characteristics, color, water activity, hardness, shrinkage, rehydration, microstructure, nutritional content, antioxidant capacity, and energy consumption of apricot slices (*p* < 0.05). (2) In terms of drying characteristics, PVD required the shortest time and resulted in the fastest drying rate; VFD required the longest time and exhibited the slowest drying rate. (3) In terms of microstructure, a honeycomb structure appeared in the apricot slices during the VFD process, with evenly arranged holes. Larger holes appeared in the apricot slices during the PVD process, accompanied by collapse. Some irregularities appeared in the apricot slices during the IRD process. Regular holes were observed during the HAD process; apricot slices were encrusted and holes collapsed, forming a dense structure. (4) In terms of quality, VFD apricot slices had the smallest color difference, the highest color saturation, the lowest degree of browning and water activity, the smallest shrinkage rate, the best rehydration ability, a high nutrient retention rate, and the strongest antioxidant capacity. The quality of apricots dried by PVD ranked second. (5) In terms of energy consumption, VFD showed the highest energy consumption, PVD showed the second-highest energy consumption, and infrared drying showed the lowest energy consumption. In summary, among the four drying methods, VFD resulted in the best quality of dried apricots and was the preferred method for producing dried apricots. However, while considering efficiency, cost, and quality, PVD is a feasible alternative to producing high-quality dried apricots. This study provides important information for selecting appropriate apricot drying methods aimed at minimizing drying time and energy consumption, and preserving product quality.

## Figures and Tables

**Table 1:** Effects of different drying methods on the color and browning degree of apricot slices.

Category	Fresh Sample	HAD	IRD	PVD	VFD
	** 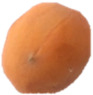 **	** 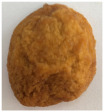 **	** 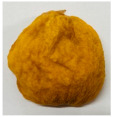 **	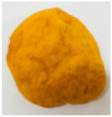	** 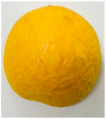 **
*L*	65.60 ± 0.98 ^b^	43.56 ± 2.16 ^e^	50.82 ± 0.95 ^d^	54.27 ± 0.44 ^c^	70.29 ± 0.84 ^a^
*a*	17.54 ± 1.14 ^c^	19.45 ± 1.18 ^c^	28.67 ± 0.89 ^a^	25.47 ± 0.96 ^b^	24.02 ± 1.25 ^b^
*b*	47.19 ± 1.49 ^c^	33.23 ± 3.19 ^d^	46.49 ± 1.24 ^c^	52.26 ± 0.86 ^b^	58.07 ± 1.54 ^a^
∆E	-	26.45 ± 1.36 ^a^	18.72 ± 0.41 ^b^	14.89 ± 0.50 ^c^	13.64 ± 0.55 ^c^
*C*	50.35 ± 1.48 ^d^	38.52 ± 3.06 ^e^	54.62 ± 1.49 ^c^	58.15 ± 0.39 ^b^	62.84 ± 1.87 ^a^
Browning degree (AU/g)	-	1.54 ± 0.14 ^a^	0.89 ± 0.67 ^b^	0.50 ± 0.08 ^c^	0.35 ± 0.04 ^c^

Values with different letters in each column are considered significantly different (*p* < 0.05).

**Table 2:** Energy consumption ratio of dried apricot slices using different drying methods.

Category	HAD	IRD	PVD	VFD
Drying time (h)	21.39 ± 1.10 ^b^	17.54 ± 0.73 ^c^	15.53 ± 0.66 ^d^	34.64 ± 1.02 ^a^
Energy consumption (kWh/kg)	5.19 ± 0.21 ^b^	3.47 ± 0.29 ^c^	4.57 ± 0.41 ^bc^	26.88 ± 1.07 ^a^
Water content (%)	14.60 ± 0.32 ^a^	14.48 ± 0.34 ^a^	14.53 ± 0.23 ^a^	14.51 ± 0.36 ^a^

Values with different letters in each column are considered significantly different (*p* < 0.05).

## Data Availability

The original contributions presented in the study are included in the article, further inquiries can be directed to the corresponding authors.
